# Incidence of Patellar Desmopathy in the Modified Maquet Technique with and without PRGF

**DOI:** 10.3390/vetsci9040180

**Published:** 2022-04-10

**Authors:** Victoria Valiño-Cultelli, Óscar Varela-López, Antonio González-Cantalapiedra

**Affiliations:** Department of Anatomy, Animal Production and Veterinary Clinical Sciences, Veterinary Faculty, Universidad de Santiago de Compostela, 15705 Lugo, Spain; oscar.varela@usc.es (Ó.V.-L.); antonio.cantalapiedra@usc.es (A.G.-C.)

**Keywords:** MMT, TTA, dog, PRGF, desmopathy, patellar ligament

## Abstract

Patellar desmopathy can lead to desmitis, discomfort, and lameness. In the traditional MMT, a pin is used for the fixation of the fragment of the osteotomized tibia to the implant and the tibial diaphysis; this pin needs to be placed below the insertion of the patellar ligament to avoid damaging it. Considering the differences between TTA and MMT, the authors consider it important to determine the incidence of patellar desmopathy in the MMT. This is a prospective study that enrolled 53 owned dogs that underwent MMT and were followed up by a complete examination and radiograph controls to establish the patellar tendon thickening and the presence of clinical desmitis. The PRGF were administrated to 29 of those patients, in order to determine if PRGF’s action could decrease ligament desmitis. The conclusions in this study were that the desmopathy produced by the MMT is similar to that generated by the TTA if the pin is carefully placed; in addition, the PRGF did not decrease the inflammation and the thickening of the ligament.

## 1. Introduction

Cranial cruciate ligament rupture (RCrCL) is one of the most common stifle joint disorders diagnosed in dogs [1,2,3] and the most frequent cause of secondary degenerative arthrosis, pain, and limps in the knee joint [4,5]. The treatment for RCrCL is surgical, and the objective is to stabilize the stifle joint by neutralizing the tibiofemoral shear forces [6]. Among these surgical techniques, there is the Tibial Tuberosity Advancement (TTA), which neutralizes cranial tibiofemoral shear force by advancing the insertion of the patellar ligament until it is perpendicular, in a 90º angle, to the tibial plateau with the joint in extension [7]; this advancement increases the distance between the tibiofemoral contact area and the insertion of the patellar ligament at the tibial tuberosity [8]; this longer lever arm results in lower forces required extending the stifle and less stress on the patellar ligament [9]. Due to this, at first, it was believed that the TTA technique could not affect the patellar ligament thickness, so the patellar desmopathy was a complication reserved for the Tibial Plateau Osteotomy (TPLO) technique [10,11,12] until 2011, when Kuhn proved that TTA could induce patellar desmopathy [13]. After that, other authors focused on examining this complication in the TTA technique [14,15]. The importance described for the patellar desmopathy is that it can lead to desmitis, discomfort, and lameness [15].

The Modified Maquet Technique (MMT) has the same functional basis as the TTA, but with a different kind of surgical procedure and different implants that support the fixation of the tibial osteotomy advancement; the MMT was developed to reduce the amount of implants in order to preserve soft tissues and associated vascularity to encourage biologic repair of the osteotomy [16]. However, in the traditional MMT, a pin is used for the fixation of the fragment of the osteotomized tibia to the implant and the tibial diaphysis; this pin needs to be placed below the insertion of the patellar ligament to avoid damaging it [17]. Considering these differences between TTA and MMT, the authors consider it important to determine the incidence of patellar desmopathy in the MMT.

To the authors’ knowledge, only one study compares the incidence of patellar desmopathy between the TTA and a type of TTA mixed with MMT, for which they used TTA implants [18], so they did not follow the traditional MMT.

Plasma Rich in Growth Factors (PRGF) is an autologous platelet concentration, obtained in a specific way described by Anitua (2009) [19], who proved it played a role in the angiogenesis, antimicrobial, analgesic, and anti-inflammatory process [20,21,22]; in addition, PRGF is a cheap and accessible source in veterinary medicine.

Taking this into account, our aim was to determine the incidence of patellar desmopathy in the MMT, our hypothesis being that the MMT can lead to the same degree or a higher degree of patellar desmopathy than those described for the TTA technique. Another objective is to determine whether the locally administered PRGF in the area can lead to developing a minor desmopathy.

## 2. Materials and Methods

### 2.1. Clinical Trial

This study was performed at the Rof Codina University Veterinary Hospital—Santiago de Compostela University (Lugo, Spain), from December 2017 to July 2020. It includes 53 skeletally mature patients who were operated with MMT and the PLA scaffolds. Twenty-four of their patients were in the control group, without PRGF (18 finished the study), and 29 patients made up the PRGF group (17 finished the study). All the patients that did not finish the study were monitored by phone call.

One of the aims of this study was to perform a monitoring of the patellar ligament thickness in a long period of time, following the recommendations of other authors that previously studied the patellar ligament desmopathy in short periods of time [13,14,15]. This is the reason why the patients that did not finish the study were excluded for the final results.

The MMT was proposed as a technique of choice because this study was designed to also determine the osteoconductive properties of a PLA scaffold [23,24], and the MMT provides a minor amount of implants per patient [23]; the PLA scaffold was fixed with a 1.5 mm diameter pin in all cases and a tension band wire (for avoiding the avulsion of the tibial tuberosity, which is a frequent complication [6]). This technique was performed in 53 owned dogs; the complete surgery process and PRGF’s fabrication and administration were described previously [23,24].

The assignation to PRGF group or control group were aleatory and blinded to the surgeons that performed the post-surgery traumatological revision at the different follow-ups. Once the osteotomy was performed, as is described in a previous paper from the authors [23,24], a total volume of 1 mL of autologous liquid PRGF were applied in the implant and 1 mL above the surrounding tissues (including the patellar ligament); another 1 mL of autologous PRGF clot was placed in the osteotomy gap. The administration of PRGF was performed only at surgery time.

The anaesthetic and analgesic protocol and the antibiotic therapy used in the surgery were published in a previous paper from the authors [23].

All the patients received the same discharge protocol: meloxicam 0.1 mg/kg PO q24h for 9 days, cefazoline 22 mg/kg PO q8h for 10 days, and digestive protection (depending on patient’s weight) for 10 days. In addition, a Robert–Jones bandage was applied from the day of the surgery up to 4 days after, and limited exercise was recommended until follow-up examination.

### 2.2. Data Collection

The follow-up was carried out by a complete physical examination and radiographs at 1 (T-1), 2 (T-2), and 5 months (T-3) after the surgery. All the radiographs were obtained using the same X-ray equipment, in laterolateral and caudocranial views, the laterolateral view being taken with the stifle at a 135° angle. The completion of all the follow-ups was needed for the inclusion criteria.

The data collected were gender, age, body weight, breed, lifestyle, level of exercise difficulty presented by the patient, dates of follow-up radiographs, scaffold size, PRGF application or not, and complications. The data of the PRGF administration were blinded for the owners and follow-up evaluators.

When complications occurred, they were assessed as major or minor complications according to a previous publication [25].

The tension band wire removal, which was previously planned in case the patient needed it, was not taken into consideration as a complication (except in the case that it was broken). Though the pin extraction was also contemplated as a possibility, it was considered as a complication related to the possible cause of the ligament thickening and desmopathy.

### 2.3. Radiographic Evaluation

The patellar ligament thickness was recorded in the laterolateral radiographs at 135º, being assessed with the open-source software OsiriX MD 11.0 (PIXMEO SARL, Geneva, Switzerland) (open-source software; www.osirixviewer.com, accessed date: 15 February 2022).

Pre-operative radiographic measurements (T-0) were performed, in order to obtain a reference measurement and have a clearer idea of the patient’s evolution, and the radiographs obtained subsequently were measured in the different follow-ups: first follow-up at 1 month (T-1), second follow-up at 2 months (T-2), and third follow-up at 5 months (T-3). The criteria for the selection of these time periods for radiograph follow-ups were based on monitoring the osteotomy healing and implants’ stability, as is recommended [8]; the first two follow-ups were according to the time that selected other authors for monitoring tendon thickness [13,14,15].

**Figure 1:**
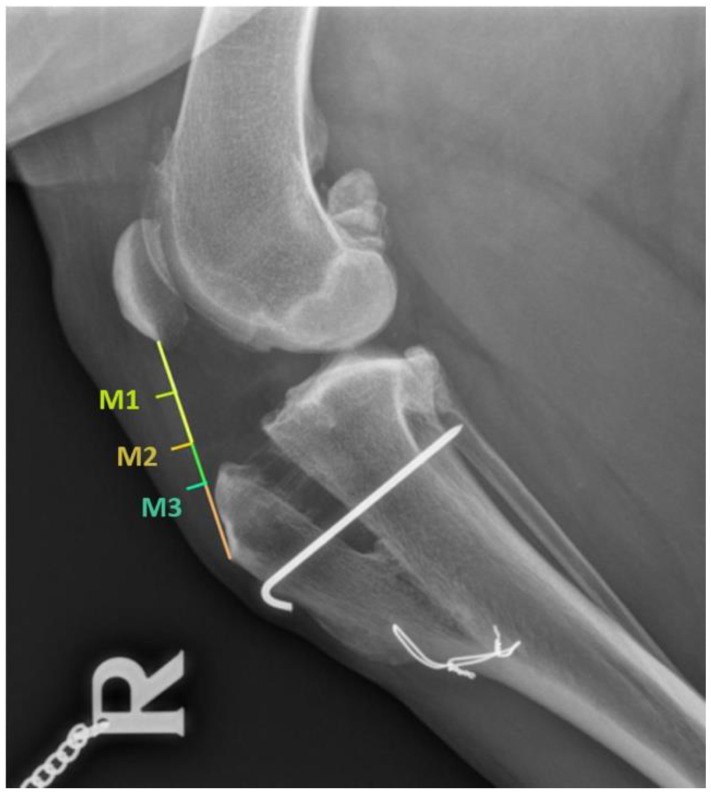
Patellar ligament measurements.

Firstly, we measured the patellar ligament total length, from its origin on the distal aspect of the patella to its insertion on the proximal aspect of the tibial tuberosity. The ligament thickness was measured at 1 cm distal to its origin, 1 cm proximal to its insertion, and the third measurement was taken at the middle of the ligament, obtained by dividing its total length into two (Figure 1).

All the radiographs were blinded for the two reviewers, and an aleatory number was assigned for each one; they were also examined for complications.

### 2.4. Desmopathy Diagnosis

The diagnosis for desmopathy is based on ultrasound, radiographic, and clinical changes [11,12,13,14,15]. An increase in the ligament thickness, taking as reference the pre-operative ligament thickness (T-0), can be considered as desmopathy [11]. In terms of symptoms, the desmopathy can be demonstrated by desmitis, discomfort, and lameness [15]; the desmitis can be detected in the patient’s traumatological exploration of the patellar ligament, finding tendon thickening and discomfort or pain at the palpation.

One of the limitations of this study is the absence of ultrasound examination of the patellar ligament.

In addition, a progressive degeneration of the patellar ligament is possible in patients undergoing TTA or MMT, and it can be detected due to ligament failure and the presence of drawer motion in the patient’s knee exploration [14].

### 2.5. Statistical Method

The statistical analysis was carried out with Sigma Plot 12.5 (Systat Software Inc., San Jose, CA, USA). The results were expressed as a mean ± standard deviation.

The analysis of the results for the tendon thickness obtained in the two groups at the different follow-ups were compared by an ANOVA test (*p* < 0.05).

## 3. Results

The descriptive statistical analysis for the follow-up days, age, weight, and the selected scaffold size were previously described and discussed [23,24].

For an easier analysis of the results, we divided the patients into two groups: the control group that had not received PRGF, and the PRGF group where the PRGF was administrated. We also divided the follow-ups: pre-surgery (T-0), first follow-up at 1 month (T-1), second follow-up at 2 months (T-2), and third follow-up at 5 months (T-3). Moreover, the measurements were divided according to the place where we measured the ligament thickness in: 1 cm distal to its origin (M-1), at the middle of the ligament (M-2), and 1 cm proximal to its insertion (M-3).

The means and standard derivations are shown below in Table 1.

Attending to perform a complete statistical analysis of the results, the data obtained were analyzed in different ways.

First, we compared the values between the different measurements M-1, M-2, and M-3 at the same time and, for the same group, and the results had no statistically significant differences between the measurements at the same time for all the follow-ups in the same group (*p* < 0.05). This was the case for both groups.

Next, we compared all the values within the same group. For the control group, there were statistically significant differences between T-1(M-2) vs. T-0(M-1); T-0(M-2); T-0(M-3); T-3(M-1), and T-3(M-2) (*p* < 0.05). For the PRGF group, there were statistically significant differences between T-1(M-2) vs. T-0(M-1); T-0(M-2); T-0(M-3), and T-3(M-1); also between T-1(M-3) vs. T-0(M-1); T-0(M-2); T-0(M-3); T-3(M-1); T-3(M-2), and T-3(M-3); as well as between T-1(M-3) vs. T2(M-1) and T-3(M-3); and finally between T-2(M-3) vs. T-0(M-1); T-0(M-2) and T-0(M-3); for all values, the significance level was *p* > 0.05.

Finally, we compared the values of both groups, and the results showed no statistically significant differences between the values for the same follow-up (*p* > 0.05).

In addition, 5.7% (2) of the patients in the control group did not show any signs of desmopathy between T-0 and T-1. At the final follow-up T-3 (5 months), 40% of the patients had no signs of desmopathy, out of whom 8.5% (3) were from the PRGF group and 31.5% (11) were from the control group.

Regarding complications, in 4 patients (7.5%), the pin was removed, out of whom three were from the PRGF group and one was from the control group, although the removal of the pin was in all cases at the owner’s request, and it did not cause any lameness or other apparent problems. The total amount of complications presented with this technique in the study is described in a previous publication [24], and there were 10 out of 53 patients (18.8%) that presented complications, five being classified as major and five as minor, according to a previous publication by Cook [25].

## 4. Discussion

The present study evaluated the presence of desmopathy in MMT and was performed from December 2017 to July 2020 in the Rof Codina University Veterinary Hospital. This study involved 53 owned dogs, out of whom 35 finished the complete study. The patients that finished the study were 17 from PRGF group and 18 were from the control group; the patients that did not complete the follow-ups were monitored by phone call. 

As mentioned above, the results about the implant function and osseointegration were previously published [23,24].

Even though it was believed that, in the TTA technique, desmopathy was a rare complication because of its biomechanics, which causes less stress on the patellar ligament [9], other later studies proved that this is a frequent sequela [13,14,15].

According to the results obtained in this study in both groups, statistically significant differences were found between T-0 (pre-surgery) and T-1 (first follow-up at one month) in one or more measurements, with the corresponding total means of 2.90 vs. 5.09 mm for the control group and 2.66 vs. 4.74 mm for the PRGF group. The mean values for T-0 and T-1 were similar to those described by Kuhn (2011) [13] with the difference that they did not find any statistically significant differences between T-0 and T-1. In contrast, our results for T-0 were lower than those described by Pettitt at the same follow-up time and were higher for T-1 [14]. In this study, the authors did not describe any statistically significant differences between T-0 and T-1, but they found that 50% of the patients had no signs of patellar ligament desmopathy following surgery at six weeks. On the other hand, Kuhn described that 100% of the patients had signs of desmopathy [13], agreeing with DeSandre-Robinson, who described 100% of desmopathy at six weeks post-surgery [15]. In our study, only 5.7% (2) of the patients, who were from control group, had no signs of patellar desmopathy after surgery at T-1. Furthermore, DeSandre-Robinson obtained mean values similar to those in our study for T-0 and T-1, and they also found statistically significant differences between these two groups [15].

The increase in the mean values between T-0 and T-1 is an expected change in patellar ligament thickness due to surgery trauma, larger tibial advancement, altered insertion angle of the patellar ligament, or postoperative activity of the dog [10,12]. In addition, it has been suggested that the arthrotomy during the surgery for the meniscus exploration could cause more desmopathy because of the parapatellar incision and the hard retraction of the patellar ligament [13,15]; this is one of the reasons why in our study we decided to avoid this practice.

**Figure 2:**
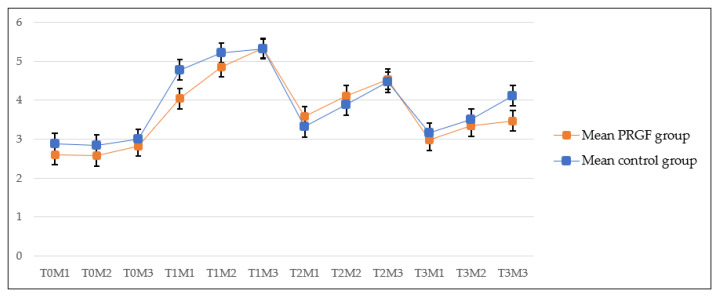
Means and standard derivations of patellar ligament thickening at the different follow-ups.

The PRGF and control groups showed statistically significant differences between T-1 and T-3, but not for all measurements, as shown in Table 1. These results are similar to those described by Kuhn [13], who had measured the ligament thickness for 16 weeks (4 months). In our study, the tendency was the same (Figure 2) between the first and the final follow-up, the ligament thickness tended to decrease, but the final thickness is greater than the initial thickness measured before the surgery [13]. This also agrees with the results published by DeSandre-Robinson [15]. In summary, the tendency in both groups is to increase the ligament thickness between pre-surgery evaluation and the first follow-up, at one month, and to decrease it between the first follow-up and the final follow-up at five months, which tally with the results reported by other authors [13,14,15].

The results of the PRGF group showed statistically significant differences between T-1(M3) vs. T2 (M-1), which indicates a decreasing tendency in ligament thickness, as shown in Graphic 1, and agrees with other authors in this regard [13,14,15]. Another difference from the control group is that T-2 (M-3) showed a statistically significant difference in all the measurements of T-0 (Table 1), as described by another author, and, after surgery, the tendency of the ligament is to increase its thickness even more near the insertion zone [15]. At the final follow-up (5 months), 40% of the patients had no signs of desmopathy, out of whom 8.5% (3) were from the PRGF group and 31.5% (11) were from the control group.

Retallack, in a study where they merged the TTA and MMT techniques, using the MMT as a basis with the plate of TTA, evaluated the ligament thickness compared to the normal ligament thickness in TTA. They agreed with the increase of ligament thickness that was described by other authors, as mentioned above. The same as in our study, they did not find any differences in ligament thickening between the MMT modified technique which they used and conventional TTA [18].

Consistent with their earlier points, our results confirmed our first hypothesis that, with MMT, the desmopathy would be the same or more severe than with TTA, our results being similar to those previously published about ligament thickness using TTA [13,14,15]. The desmopathy produced by the MMT is similar to that generated by the TTA technique; one of our concerns were that, using the MMT, the desmopathy could be more pronounced than with the TTA due to pin placement, but, if the pin was carefully placed, it did not increase the ligament desmopathy.

There were no statistically significant differences for ligament thickness between the PRGF and the control group. This rejects our second hypothesis that PRGF, in the way that we used them, decrease the inflammation and the ligament thickening after surgery in dogs.

Platelets play an important role in primary haemostasis, promoting angiogenesis [26] and recruitment of mesenchymal stem cells [27], inducing a complex inflammatory response at the injury site [28] and are a source of growth and differentiation factors [29]. These PRP growth factors contribute to macrophage recruitment, angiogenesis, chemotaxis of keratinocytes, and the mitogenic activity of fibroblasts [21].

This is the reason why the PRP plays a significant role in angiogenesis, as well as the antimicrobial, anti-inflammatory, and analgesic process [20,21,22], turning them into an excellent alternative in soft tissue regeneration.

The use of the PRGF had proven its efficacy in tendon and ligament repair in humans and laboratory animals in multiple studies [30,31,32,33,34], although, to the best of the authors’ knowledge, there are no studies that evaluate the PRGF action in the tendon and/or ligament healing in dogs. We decided to use the PRGF (that is a type of PRP obtained with a standardized protocol) because it is known that the efficacy of PRP can be affected by the different platelet concentration due to the variety of the PRP protocols, by the type of injury, application, and/or the animal species [20,35,36,37]; and this decision could avoid the first variable, but the other ones could justify the reason why in our study the PRGF used did not show any statistically significant results.

Despite the fact that the local administration of the PRP is not the most common practice, previous studies in humans and animals demonstrated beneficial results with its topical administration for wound management [21,38,39,40,41,42]; moreover, the local application of the PRP obtained good results in other fields: orally to improve gastric ulcers in rats [43], improving dry eye symptoms in humans [44], and in medial collateral ligament tear on rabbits [32], even with a single application [32].

An issue that concerned us was the fact that PRP, through the activation of angiogenesis, may cause oedema, discomfort, swelling, and inflammation [45,46], but none of these symptoms were detected in our patients in an atypical way by orthopaedic exploration or in the radiographs, compared to the control group.

It is important to keep in mind that this study was developed to evaluate the results over a long period of time, and the administration of NSAIDs is a limitation that could decrease the inflammation of the ligament, whereas the decision for its administration was based on ethics, to relieve patients’ pain during the first days.

It is believed that the TTA plate plays an important role in the development of patellar desmopathy after using the TTA technique, especially when the plate is over or under bending [13]. However, in our study, in which MMT did not require any plate, the desmopathy was generated in the same degree as previous studies that measured the ligament thickness in the conventional TTA, thus we can assume that the placement of the plate does not influence the appearance of desmopathy.

According to previous studies, the clinical impact of patellar desmopathy appears to be of questionable significance; it is assumed that the patellar ligament thickening is a physiological response to tibial tuberosity advancement [13,14]. Taking into account our results about lameness using the MMT, published in a previous paper [24], it seems that the patellar desmopathy did not have an important role in the clinical outcome. The percentage of lameness presented in the aforementioned study was within a normally expected range, according to previous publications, and it is considered that the role played by the muscular atrophy of the patient in the development of the lameness is more important [23,24,47,48]. In any case, as recommended by other authors [13,14,15], in the orthopedic exam, the zone of the ligament projection was palpated, and the patients did not show evidence of pain.

Complications derived from the MMT were described in a previously published paper [24]; there were 10 out of 53 patients (18.8%) who presented complications, this percentage being within the range of values presented in other papers that studied the TTA (11–31.5%) [6,49,50,51]. Five were classified as major and five as minor complications [25]. Regarding ligament thickening, our concern was the placement of the pin, which is close to the insertion of the patellar ligament and could damage it. In four patients (7.5%), the pin was removed, out of whom three were from the PRGF group and one was from the control group, although the removal of the pin was in all cases at the owner’s request and it did not cause any lameness or other apparent problems. The patients did not present any pain during ligament palpation at the orthopedic examination. Taking this into account, we can state that, if the pin is carefully placed, below the insertion of the patellar ligament, it should not cause any problems, although a study over a longer time period should be conducted to support this statement.

No complications associated with the use of the PRGF were observed.

Some authors mention the possibility of a progressive degeneration of the patellar ligament [14], but this is a complication that was not observed in our study, at least during the five-month follow-up.

The limitations of the present study are the lack of histology that could support the absence of statistically significant results with PRGF and the impossibility to perform a more accurate imaging technique that could provide us with more information, such as magnetic resonance or echography.

## 5. Conclusions

The desmopathy produced by the MMT is similar to that generated by the TTA technique. The tendency between pre-surgery and the first month post-surgery is to increase the ligament thickness and to decrease between the first month post-surgery and the five months post-surgery.

The PRGF, in the way that we used them, did not decrease the inflammation and the thickening of the ligament after surgery in dogs. However, the PRGF did not lead to any complication.

Considering that we did not use a plate, according to the MMT, and our results demonstrated that desmopathy may appear despite this fact, the plate used in the TTA technique is apparently not related to the desmopathy as other authors believed, although more studies are needed to confirm this hypothesis.

If the pin is carefully placed, below the insertion of the patellar ligament, it should not cause complications, although a study over a longer time period should be carried out to support this statement.

## Figures and Tables

**Table 1:** Means and standard derivations of patellar ligament thickening at the different follow-ups.

CONTROL GROUP (*n* = 17) (mm)												
	Pre-surgery (T-0)			First follow-up (T-1)			Second follow-up (T-2)			Third follow-up (T-3)		
	M-1	M-2	M-3	M-1	M-2	M-3	M-1	M-2	M-3	M-1	M-2	M-3
	2.88 ± 0.73	2.84 ± 0.78	2.99 ± 0.81	4.77 ± 2.48	5.21 ± 2.50	5.31 ± 2.68	3.31 ± 1.48	3.88 ± 1.74	4.46 ± 1.83	3.15 ± 1.42	3.50 ± 1.58	4.11 ± 2.21
Mean	2.90			5.09			3.88			3.58		
PRGF GROUP (*n* = 18) (mm)												
	Pre-surgery (T-0)			First follow-up (T-1)			Second follow-up (T-2)			Third follow-up (T-3)		
	M-1	M-2	M-3	M-1	M-2	M-3	M-1	M-2	M-3	M-1	M-2	M-3
	2.59 ± 0.73	2.57 ± 0.63	2.82 ± 0.56	4.03 ± 1.06	4.86 ± 2.27	5.33 ± 2.50	3.58 ± 1.05	4.10 ± 1.39	4.53 ± 1.68	2.96 ± 0.87	3.33 ± 1.31	3.46 ± 0.91
Mean	2.66			4.74			4.07			3.25		

## Data Availability

Not applicable.
